# Nanoprobes for Tumor Theranostics

**DOI:** 10.3390/bios12111022

**Published:** 2022-11-16

**Authors:** Jiaoyang Zhu, Zheyu Shen

**Affiliations:** School of Biomedical Engineering, Southern Medical University, 1023 Shatai South Road, Guangzhou 510515, China

This Special Issue of *Biosensors*, entitled “Nanoprobes for Tumor Theranostics”, aims to report the research progress of using nanoprobes for the diagnosis and therapy of tumors, and promote their applications. We hope to attract the attention of researchers from various disciplines of chemistry, materials science, oncology, biology, and medicine. Currently, numerous scientific challenges in tumor theranostics remain, which must be surmounted to improve human health and quality of life. This Special Issue aims to contribute to furthering creative progress in the field via publishing ten papers reporting the synthesis, applications, and advances of several nanoprobes for tumor theranostics.

Circulating tumor cells (CTCs), released from tumor sites into the peripheral blood, have been recognized as promising biomarkers for cancer prognosis, treatment monitoring, and metastasis diagnosis. However, the number of CTCs in the peripheral blood is low, making it a technical challenge to isolate, enrich, and identify CTCs from patient blood samples. Yue Pan et al. [1] developed a simple, effective, and inexpensive strategy to capture and identify CTCs from the blood samples of patients with ovarian cancer (OC) using the folic acid (FA) and antifouling-hydrogel-modified fluorescent magnetic nanoparticles. The hydrogel showed a good antifouling property against peripheral blood mononuclear cells (PBMCs). The FA was coupled to the hydrogel surface as the targeting molecule for CTC isolation, maintained a good capture efficiency for SK-OV-3 cells (95.58%), and successfully isolated 2–12 CTCs from 10 OC patients’ blood samples. The FA-modified fluorescent magnetic nanoparticles were thus successfully used for the capture and direct identification of CTCs from the blood samples of OC patients.

Accurate detection of multiple analytes from a single measurement, requiring complex encoding systems, is critical in modern bioanalysis. Umar Azhar et al. developed a novel bioassay with Raman-coded antibody supports (polymer microbeads with different Raman signatures) and surface-enhanced Raman-scattering (SERS)-coded nanotags (organic thiols on a gold nanoparticle surface with different SERS signatures) as a model fluorescent, label-free, bead-based multiplex immunoassay system [2]. The developed homogeneous immunoassays included two surface-functionalized monodisperse Raman-coded microbeads of polystyrene and poly(4-tert-butylstyrene) as the immune solid supports, and two epitope-modified nanotags (self-assembled 4-mercaptobenzoic acid or 3-mercaptopropionic acid on gold nanoparticles) as the SERS-coded reporters. Such multiplex Raman/SERS-based microsphere immunoassays could selectively identify specific paratope–epitope interactions from one sample solution under the illumination of a single laser, and thus hold great promise for future suspension multiplex analysis in a diverse range of biomedical applications.

Cell-based immunotherapy has become one of the most promising ways to completely eliminate cancer. The major challenge is to effectively promote a proper immune response, inducing T cells to kill the cancer cells. Huan Chen et al. [3] investigated the effect of T-cell-mediated immunotherapy trigged by Au DENPs-MPC (zwitterion 2-methacryloyloxyethyl phosphorylcholine (MPC)-functionalized dendrimer-entrapped gold nanoparticles) loading oli-godeoxynucleotides (ODNs) of an unmethylated cytosine guanine dinucleotide (CPG). They first synthesized Au DENPs MPC, evaluated its capability to compress and transfect CpG-ODN to bone marrow dendritic cells (BMDCs), and investigated the potential of using T cells stimulated by matured BMDCs to inhibit tumor cell growth. The developed Au DENPs-MPC could apparently reduce the toxicity of Au DENPs, and enhanced transfer of CpG-ODN to the BMDCs to stimulate maturation, as demonstrated by the 44.41–48.53% increase in different surface maturation markers. The transwell experiments certificated that ex vivo-activated T cells display excellent antitumor properties, effectively inhibiting the growth of tumor cells. These results suggest that Au DENPs-MPC can deliver CpG-ODN efficiently to enhance the antigen presentation ability of BMDCs to activate T cells, indicating that T-cell-based immunotherapy mediated by Au DENPs-MPC loaded with CpG-ODN is an exceedingly promising treatment for cancer.

Diverse drug loading approaches for human heavy-chain ferritin (HFn), a promising drug nanocarrier, have been established. However, the antitumor drug loading ratio and protein carrier recovery yield are bottlenecks for future clinical application. The mechanisms behind drug loading have not been elaborated. Shuang Yin et al. [4] introduced a thermally induced approach to loading the antitumor drug doxorubicin hydrochloride (DOX) into HFn and two functionalized HFns, HFn-PAS-RGDK, and HFn-PAS. Optimal conditions were obtained through orthogonal tests. All three HFn-based proteins achieved a high protein recovery yield and drug loading ratio. Size exclusion chromatography (SEC) and transmission electron microscopy (TEM) results show the majority of DOX-loaded protein (protein/DOX) retained its nanocage conformation. Computational analysis, molecular docking followed by molecular dynamic (MD) simulation, revealed the mechanisms of DOX loading and the formation of by-products by investigating noncovalent interactions between DOX and the HFn subunit and possible binding modes of DOX and HFn after drug loading. In in vitro tests, DOX in protein/DOX entered the tumor cell nucleus and inhibited tumor cell growth.

Living-sample viability measurement is an extremely common process in medical, pharmaceutical, and biological fields, especially drug pharmacology and toxicology detection. Nowadays, there are a number of chemical, optical, and mechanical methods that have been developed in response to the growing demand for simple, rapid, accurate, and reliable real-time living-sample viability assessment. In parallel, the development trend of viability measurement methods (VMMs) has increasingly shifted from traditional assays towards the innovative atomic force microscope (AFM) oscillating sensor method (referred to as nanomotion), which takes advantage of the adhesion of living samples to an oscillating surface. A comprehensive review of the most common VMMs, laying emphasis on their benefits and drawbacks, as well as evaluating their potential utility, was provided by Hamzah Al-madani et al. [5] In addition, they discussed the nanomotion technique, focusing on its applications, sample attachment protocols, and result display methods. Furthermore, challenges and future perspectives with regard to nanomotion were commented on, mainly emphasizing scientific restrictions and development orientations.

The recent development of ion interference therapy (IIT) based on inorganic nanoparticles was introduced by Yongjie Chi et al. [6] They summarized the advantages and disadvantages of this treatment and the challenges of future development, hoping to provide a reference for future research. As an essential substance for cell life activities, ions play an important role in controlling the cell osmotic pressure balance, intracellular acid–base balance, signal transmission, biocatalysis, and so on. The imbalance of ion homeostasis in cells can seriously affect cells activities, cause irreversible damage to cells, and induce cell death. Therefore, artificially interfering with the ion homeostasis in tumor cells has become a new means by which to inhibit the proliferation of tumor cells. This treatment is called IIT. Although some molecular carriers of ions have been developed for intracellular ion delivery, inorganic nanoparticles are widely used in ion interference therapy because of their greater capacity for ion delivery and superior biocompatibility compared with molecular carriers.

As an emerging and powerful material, aggregation-induced emission luminogens (AIEgens), which can simultaneously provide precise diagnosis and efficient therapeutics, have exhibited significant superiorities in the field of phototheranostics. Of particular interest is phototheranostics based on AIEgens with emissions in the second near-infrared (NIR-II) range (1000–1700 nm), which has promoted the feasibility of their clinical applications by virtue of numerous preponderances benefiting from the extremely long wavelength. Yonghong Tan et al. [7] summarized the past 3 years of advances in the field of phototheranostics based on NIR-II AIEgens, including the strategies of constructing NIR-II AIEgens and their applications in different theranostic modalities (FLI-guided PTT, PAI-guided PTT, and multimodal-imaging-guided PDT–PTT synergistic therapy); in addition, a brief conclusion including perspectives and challenges in the field of phototheranostics is provided at the end.

Smart nanomedicines that are capable of diagnosis and therapy (theranostics) in one-nanoparticle systems are highly desirable for improving cancer treatment outcomes. Magnetic nanoplatforms are ideal for cancer theranostics, because of their diverse physiochemical properties and biological effects. In particular, a biocompatible iron oxide nanoparticle-based magnetic nanoplatform exhibits multiple magnetic-responsive behaviors under an external magnetic field and can realize the integration of diagnosis (magnetic resonance imaging, ultrasonic imaging, photoacoustic imaging, etc.) and therapy (magnetic hyperthermia, photothermal therapy, controlled drug delivery and release, etc.) in vivo. Furthermore, due to considerable variation among tumors and individual patients, iron oxide nanoplatforms, designed via the coordination of diverse functionalities for efficient and individualized theranostics, are urgently needed. Wangbo Jiao et al. [8] presented an up-to-date overview on iron oxide nanoplatforms, including both iron oxide nanomaterials and those that respond to an externally applied magnetic field, with an emphasis on their applications in cancer theranostics.

Second near-infrared (NIR-II) fluorescent imaging has been widely applied in biomedical diagnosis due to its high spatiotemporal resolution and deep tissue penetration. In contrast to the “always on” NIR-II fluorescent probes, activatable NIR-II fluorescent probes specifically target biological tissues, demonstrating a higher imaging signal-to-background ratio and a lower detection limit. Therefore, it is of great significance to utilize disease-associated endogenous stimuli (such as pH values, enzyme existence, hypoxia condition, and so on) to activate NIR-II probes and achieve switchable fluorescent signals for specific deep bioimaging. Dong Li et al. [9] introduced recent strategies and mechanisms for activatable NIR-II fluorescent probes and their applications in biosensing and bioimaging. Moreover, potential challenges and perspectives with regard to activatable NIR-II fluorescent probes were also discussed.

Aptamers, owing to their small size, low toxicity, good specificity, and excellent biocompatibility, have been widely applied in biomedical areas. Therefore, the combination of nanomaterials with aptamers offers a new method for cancer treatment. Liangxi Zhu et al. [10] briefly introduced this topic. They discussed the application of aptamers for the treatment of breast, lung, and other cancers. Finally, perspectives on challenges and future applications of aptamers in cancer therapy were discussed.

In summary, this Special Issue includes four research papers reporting the most recent research progress in CTC detection using fluorescent magnetic nanoparticles, the SERS-coded multiplex immunoassay system, T-cell-mediated immunotherapy, and human heavy-chain ferritin (HFn) drug nanocarriers, and six review papers reporting on living-sample viability measurement methods, IIT based on inorganic nanoparticles, NIR-II AIEgens, iron oxide nanoplatforms, activatable NIR-II fluorescent probes, and aptamers. Altogether, these papers present the most promising emerging nanoprobes in medicine and clinical research for tumor theranostics.
